# Beyond the label: real-world gastrointestinal adverse events associated with vedolizumab – a decade of FAERS pharmacovigilance

**DOI:** 10.3389/fphar.2025.1700820

**Published:** 2026-01-14

**Authors:** Jianhong Zheng, Chunfeng Qiu, Zhen Zhang

**Affiliations:** 1 Department of Pharmacy, Xinglin Hospital of Xiamen, Xinglin Branch of the First Affiliated Hospital of Xiamen University, School of Medicine, Xiamen University, Xiamen, Fujian, China; 2 Drug Clinical Trial Institution & Department of Pharmacy, The First Affiliated Hospital of Xiamen University, School of Medicine, Xiamen University, Xiamen, Fujian, China

**Keywords:** disproportionality analysis, FAERS, gastrointestinal adverse events, inflammatory bowel disease, reporting odds ratio, vedolizumab

## Abstract

**Background:**

Vedolizumab is a gut-selective biologic widely used for inflammatory bowel disease (IBD). While randomized controlled trials (RCTs) provide initial safety profiles, real-world pharmacovigilance data are crucial for identifying a broader spectrum of adverse events (AEs), including rare or underrecognized associations. This study aimed to comprehensively characterize the real-world reporting frequency and characteristics of gastrointestinal AEs associated with vedolizumab using spontaneous reporting data.

**Methods:**

We analyzed 59,976 vedolizumab-related individual case safety reports from the U.S. Food and Drug Administration Adverse Event Reporting System (FAERS) database, covering the period from Q1 2014 to Q4 2024. After meticulous exclusion of IBD diagnostic terms, 17,943 mentions of vedolizumab-associated gastrointestinal AEs were included. Disproportionality analysis, employing reporting odds ratios (ROR) with a threshold of ≥20 cases and a 95% confidence interval lower bound >1, was performed to detect significant signals. Descriptive analyses, time-to-onset (TTO) analysis, and a semi-quantitative clinical relevance assessment system were also utilized.

**Results:**

Disproportionality analysis identified 95 significant gastrointestinal AE preferred terms (PTs). Common and strongly signaled events included diarrhea, abdominal pain, haematochezia, and frequent bowel movements. Notably, 70 of these 95 significant PTs (73.7%) are not explicitly listed in the current vedolizumab drug label, suggesting potential novel or underrecognized associations. Most reported AEs were serious (90.09%). Analysis of demographic data revealed significant differences in the proportion of serious reports across gender, weight, and age groups. Males (91.44%) and patients in the <50 kg group (87.30%) had higher proportions of serious reports. Notably, adult patients (18∼65 years) showed a higher proportion of serious outcomes compared to pediatric and elderly groups, although this finding may be influenced by data completeness bias. The median time-to-onset for gastrointestinal AEs was 190 days.

**Conclusion:**

This comprehensive real-world FAERS analysis identifies both known and a substantial number of previously underrecognized gastrointestinal AEs associated with vedolizumab. The high proportion of serious events, particularly observed in male patients and those with lower body weight, underscores the need for enhanced clinical vigilance and targeted monitoring, especially early in the treatment course. These hypothesis-generating findings warrant further validation through dedicated clinical studies.

## 1 Introduction

Vedolizumab, a humanized monoclonal antibody that selectively targets the α4β7 integrin, has emerged as a pivotal therapeutic agent for individuals with moderate-to-severe inflammatory bowel disease (IBD), encompassing both ulcerative colitis and Crohn’s disease (Feagan et al., 2013; Sandborn et al., 2013). Its gut-selective immunosuppressive mechanism offers a significant advantage by minimizing systemic side effects often associated with broader-acting immunomodulators, thereby improving the benefit-risk profile for many patients (Abu-Sbeih et al., 2018). Despite its established efficacy and generally favorable safety profile demonstrated in pivotal clinical trials, concerns regarding gastrointestinal adverse events (AEs) persist, particularly as its indications expand and its utilization in real-world clinical practice grows (Ahuja et al., 2023). The foundational clinical development programs, including the GEMINI I and II trials, provided initial characterization of vedolizumab’s gastrointestinal toxicity spectrum, identifying events such as diarrhea, abdominal pain, constipation, and gastrointestinal hemorrhage (Cohen et al., 2020). However, the stringent inclusion and exclusion criteria typical of randomized controlled trials (RCTs), coupled with relatively circumscribed follow-up durations, may limit the detection of rare or delayed adverse events and restrict the generalizability of these pre-marketing safety findings to the broader, more heterogeneous patient populations encountered in routine care.

In the post-approval landscape, pharmacovigilance methodologies utilizing large spontaneous reporting systems, such as the U.S. Food and Drug Administration Adverse Event Reporting System (FAERS), have become indispensable for hypothesis generation and detecting rare, serious, or unexpected AEs that may not have been apparent during pre-marketing evaluation (Morris et al., 2024). Disproportionality analyses, particularly the calculation of reporting odds ratios (RORs), are instrumental in identifying statistically significant drug-event associations within these extensive real-world datasets, thereby complementing and extending insights derived from RCTs (Yang et al., 2024). Contemporary pharmacovigilance research has further refined risk signal management, incorporating structured scoring systems to differentiate clinically pertinent signals from statistical noise, thus enhancing the practical utility of these findings for further investigation (Mao et al., 2025). Indeed, recent explorations of FAERS and other global pharmacovigilance databases have successfully identified both established and novel gastrointestinal safety signals for various therapeutic agents, including related biologics and other drug classes like semaglutide, underscoring the dynamic and evolving nature of AE profiles as real-world experience accumulates (Osei et al., 2024).

Given vedolizumab’s widespread and growing use in diverse IBD patient populations, a comprehensive, granular assessment of its real-world gastrointestinal safety profile through large-scale pharmacovigilance is essential. While clinical trials provided foundational safety data, a detailed analysis of real-world gastrointestinal AEs reported to FAERS, complete with signal stratification by patient characteristics, a semi-quantitative assessment of potential clinical relevance, and temporal pattern analysis, is urgently needed to generate hypotheses that can refine risk assessment and guide future clinical management. This study aims to address this gap by conducting a detailed disproportionality analysis of vedolizumab-associated gastrointestinal AEs within the FAERS database, utilizing a multi-faceted pharmacovigilance approach to characterize known signals and identify potentially novel or underrecognized associations that warrant further investigation.

## 2 Methods

### 2.1 Study design and data source

This study is an observational, retrospective pharmacovigilance analysis utilizing the FAERS, which is a validated and widely used real-world pharmacovigilance resource. The approach aligns with established disproportionality methodologies for detecting potential drug-AE associations within large spontaneous reporting systems, providing a structured and statistically robust means of AE signal detection (Zou et al., 2024; Shu et al., 2022). The FAERS dataset was obtained from the FDA Quarterly Data Extract Files (https://fis.fda.gov/extensions/FPD-QDE-FAERS/FPD-QDE-FAERS.html), covering the period from the first quarter (Q1) of 2014 to the fourth quarter (Q4) of 2024. This timeframe encompasses the majority of vedolizumab’s post-marketing period and ensures a comprehensive capture of real-world reporting.

### 2.2 Data extraction and preprocessing

The FAERS database is organized into seven core relational tables: patient demographic information (DEMO), drug information (DRUG), adverse event reactions (REAC), patient outcomes (OUTC), report source (RPSR), drug therapy dates (THER), and drug indications (INDI). To ensure data integrity and avoid artificial inflation of signal counts, reports identified as deleted by the FDA were systematically removed. Deduplication was performed strictly following the latest FDA guidelines, which prioritize reports based on CASEID, FDA_DT, and PRIMARYID to retain the most relevant and recent entry for each unique case (Vestergaard Kvist et al., 2021).

Vedolizumab exposure was ascertained by searching for both its generic name (“vedolizumab”) and trade name (“ENTYVIO”) within the DRUG and PROD_AI columns. To focus the analysis on reports where vedolizumab was the primary suspect drug, we restricted reports to those marked with the role code “PS” (Primary Suspect). Adverse events were coded according to MedDRA version 27.1. We mapped all preferred terms (PTs) classified under the “gastrointestinal disorders” system organ class (SOC: 10017947). A critical step in this analysis was the exclusion of AE PTs corresponding to underlying disease diagnostic manifestations or explicit disease activity terms commonly used to describe IBD (e.g., “ulcerative colitis,” “Crohn’s disease,” “inflammatory bowel disease flare-up,” “disease progression”). This rigorous exclusion aimed to mitigate direct confounding by indication by preventing reports of IBD diagnoses themselves from being counted as drug-associated AEs (Caster et al., 2020).

It is important to note that gastrointestinal symptoms such as bleeding, perforation, and changes in bowel habits, which can be inherent to IBD pathophysiology, were not excluded, as they can also represent drug-attributable or drug-exacerbated events. However, we acknowledge that definitively distinguishing between disease-related symptoms and drug-induced adverse events in spontaneous reports without detailed clinical context remains an inherent challenge of this data source.

**FIGURE 1:**
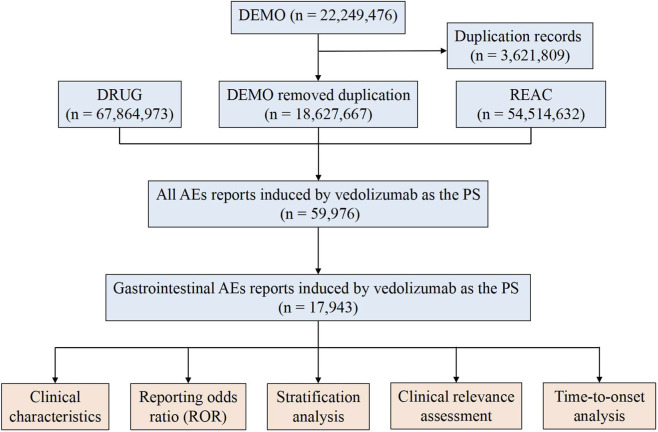
The process of selecting vedolizumab-associated gastrointestinal AEs from FAERS database. Abbreviations: AEs, adverse events.

Each report’s available clinical characteristics—gender, age, weight, outcomes, reporter type, reported country, and reporting year—were extracted. Outcomes were stratified into serious (including death, life-threatening, hospitalization, disability, congenital anomaly, required intervention, and “other serious”) or non-serious categories. It is important to note that a single report may describe multiple serious outcomes (Macaluso et al., 2023). The overall workflow for data extraction and cleaning is illustrated in Figure 1.

### 2.3 Signal detection and statistical analysis

All data extraction, cleaning, and statistical analyses were performed using R statistical software (version 4.2.3) with relevant packages for data manipulation and statistical tests (e.g., “data.table,” “dplyr,” “stats”). Disproportionality analysis was performed using the reporting odds ratio (ROR), a standard method for identifying potential drug-event associations within large pharmacovigilance databases (Shu et al., 2022). The ROR quantifies the disproportionate reporting of a specific AE in association with vedolizumab compared to its reporting with all other drugs in the FAERS database. For each PT of interest, a 2 × 2 contingency table was constructed to calculate the ROR and its 95% confidence interval (95% CI) (Supplementary Table S1). A safety signal was considered statistically significant if the lower limit of the ROR 95% CI (ROR_025_) exceeded 1 (Zhou et al., 2023). To enhance the robustness of detected signals and mitigate the influence of spurious associations driven by very low case counts, PTs with fewer than 20 associated vedolizumab reports were excluded from the disproportionality analysis (Cecco et al., 2024). It is important to note that disproportionality analysis primarily identifies statistical associations and does not establish causality. Furthermore, given the large number of PTs analyzed, the issue of multiple testing is inherent; therefore, statistical significance in this context should be interpreted as a signal of disproportionality requiring further investigation, rather than a definitive causal link. The conservative adjustments for multiple comparisons (e.g., Bonferroni) are generally avoided in spontaneous reporting systems, as they can suppress potentially important, rare signals and are not aligned with the hypothesis-generating nature of pharmacovigilance signal detection (Caster et al., 2020).

Further statistical analysis was conducted to compare the characteristics of reports classified as serious versus non-serious. Differences in categorical variables (gender, age, weight, AE distribution) were assessed using Pearson’s chi-squared (*χ* ^2^) test or Fisher’s exact test, as appropriate. Statistical significance for all comparisons was defined as *p*-value <0.05. It is important to note that disproportionality analysis primarily identifies statistical associations and does not establish causality. Furthermore, given the large number of PTs analyzed, the issue of multiple testing is inherent; therefore, statistical significance in this context should be interpreted as a signal of disproportionality requiring further investigation, rather than a definitive causal link.

To investigate the influence of demographic factors and reporter types on the association between vedolizumab and gastrointestinal disorders at the SOC level, subgroup analyses were performed. These stratifications included gender (female vs. male), age categories (<18, 18∼45, 46∼65, and >65 years), weight categories (<50, 50∼100, and >100 kg), and reporter type (healthcare professional vs. consumer). For each subgroup, the ROR and its 95% CI were calculated to assess the persistence of disproportionate reporting.

### 2.4 Clinical relevance assessment of signal strength

Recognizing the clinical significance of detected signals, a semi-quantitative signal strength assessment system was employed to prioritize the statistically significant disproportionality signals based on their potential clinical impact (Supplementary Table S2) (Cecco et al., 2024). This prioritization system integrated metrics directly derived from the FAERS data and publicly available lists, considering four key features: the reporting frequency, the signal stability, the reported case fatality rate, the classification of the AE as an important medical event (IME) or designated medical event (DME) according to the European Medicines Agency (EMA) lists (Guo et al., 2022). A composite score was assigned to each significant PT, categorizing signals into three relevance levels: low (score 0∼2), moderate (score 3∼5), and high (score 6∼8) (Gastaldon et al., 2022).

### 2.5 Time-to-onset (TTO) analysis

The TTO for each AE was calculated as the interval, in days, between the reported start date of vedolizumab use (START_DT in the THER file) and the date of AE onset (EVENT_DT in the DEMO file) (Chen et al., 2021). Reports with illogical date entries (e.g., EVENT_DT preceding START_DT), inaccurate date formats, or missing date information were excluded from the TTO analysis to ensure data integrity. The distribution of TTO was summarized using the median and interquartile range (IQR).

The reporting frequency of AEs often varies over time, and statistical analysis of TTO uses the Weibull shape parameter (WSP) test to describe the trend of the reporting hazard over time (Ren et al., 2025; Zhan et al., 2025). The Weibull distribution was characterized by two parameters: scale (α) and shape (β). The shape parameter β provides insight into the hazard function over time: β < 1 indicates an “early failure” pattern where the hazard of occurrence decreases over time; β ≈ 1 suggests a constant hazard (“random failure”); and β > 1 indicates a “wear-out failure” pattern where the hazard of occurrence increases over time (Ren et al., 2025). The WSP test was performed for the groups of signals categorized by clinical priority (moderate and low) to understand their respective temporal profiles (Shu et al., 2022; Cecco et al., 2024).

## 3 Results

### 3.1 Descriptive analysis

Between the Q1 of 2014 and the Q4 of 2024, the FAERS database processed a total of 18,627,667 AE reports. After comprehensive deduplication and filtering to include only reports where vedolizumab was identified as the primary suspect drug and that contained at least one gastrointestinal AE term falling under the pre-specified SOC, 59,976 individual case safety reports were included in our analysis. These reports detailed 17,943 vedolizumab-associated gastrointestinal AE mentions. The clinical characteristics of the patients and reports are summarized in Table 1. Among the reports with specified gender (n = 17,564), females constituted a slightly larger proportion (n = 10,306, 58.68%) compared to males (n = 7,258, 41.32%). The gastrointestinal AEs treated with vedolizumab were most frequently reported in patients aged 18∼45 years (n = 5,517, 42.02%), followed by the 46∼65 years group (n = 4,348, 33.11%). Elderly patients (>65 years) accounted for 22.47% (n = 2,950) of reports, while pediatric patients (<18 years) constituted the smallest group (n = 311, 2.37%). For the 2,012 reports containing weight data, 79.82% (n = 1,606) involved patients weighing between 50 and 100 kg. Serious outcomes were documented in a high proportion of reports (n = 16,164, 90.09%), a characteristic inherent to spontaneous reporting systems; hospitalization (n = 5,719, 31.87%) and “other serious medical events” (n = 10,070, 56.12%) were the most frequently reported serious outcomes. Death was reported in 1.24% (n = 223) of the included gastrointestinal AE cases. Reports were predominantly submitted by consumers (n = 9,000, 50.16%) and physicians (n = 4,916, 27.40%). The majority of reports originated from North America, with Canada (n = 10,743, 59.87%) and the United States (n = 3,186, 17.76%) being the most frequent reporting countries. A notable increase in the absolute number of vedolizumab-associated gastrointestinal AE reports was observed from 2021 onwards, with a rise from 1,079 reports in 2020 to 6,649 reports in 2024, likely reflecting increased drug utilization.

**TABLE 1:** Characteristics of patients with vedolizumab-associate gastrointestinal AEs from the FAERS database.

Clinical characteristics	Vedolizumab-associate gastrointestinal AEs (N = 17,943)	
	Available number	Value
Gender, n (%)		
Female	17,564 (97.89)	10,306 (58.68)
Male		7,258 (41.32)
Age (years), n (%)		
<18	13,130 (73.18)	315 (2.40)
18∼45		5,517 (42.02)
46∼65		4,348 (33.11)
>65		2,950 (22.47)
Weight (kg), n (%)		
<50	2,012 (11.21)	252 (12.52)
50∼100		1,606 (79.82)
>100		154 (7.65)
Outcomes, n (%)		
Serious outcomes	17,943 (100.00)	16,164 (90.09)
Hospitalization		5,719 (31.87)
Death		223 (1.24)
Life-threatening		84 (0.47)
Disability		65 (0.36)
Required intervention		2 (0.01)
Congenital anomaly		1 (0.01)
Other serious outcomes		10,070 (56.12)
Non-serious outcomes		1,779 (9.91)
Reporter type, n (%)		
Consumer	17,943 (100.00)	9,000 (50.16)
Physician		4,916 (27.40)
Health professional		2,062 (11.49)
Other health-professional		456 (2.54)
Pharmacist		168 (0.94)
Lawyer		3 (0.02)
Missing		1,338 (7.46)
Reported country (Top 5), n (%)		
Canada	17,943 (100.00)	10,743 (59.87)
United States		3,186 (17.76)
United Kingdom		556 (3.10)
Brazil		509 (2.84)
Australia		402 (2.24)
Reporting year, n (%)		
2014	17,943 (100.00)	31 (0.17)
2015		234 (1.30)
2016		475 (2.65)
2017		493 (2.75)
2018		742 (4.14)
2019		817 (4.55)
2020		1,079 (6.01)
2021		2,007 (11.19)
2022		2,256 (12.57)
2023		3,160 (17.61)
2024		6,649 (37.06)

Abbreviations: AEs, adverse events; n, number of cases.

### 3.2 Disproportionality analysis

**FIGURE 2:**
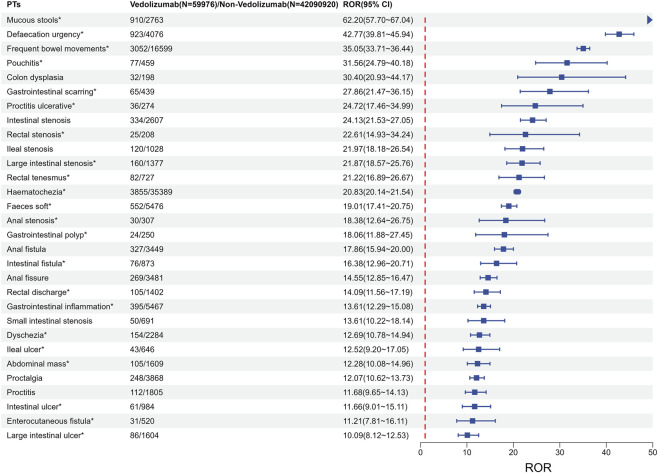
The number of reports and signal strength (ROR) for the top 30 gastrointestinal AEs. Data represents vedolizumab-associated cases compared against the full FAERS background. Notes: * Emerging findings of vedolizumab-associated gastrointestinal AEs from FAERS database. Abbreviations: PTs, preferred terms; CI, confidence interval; ROR, reporting odds ratio.

As identified in the descriptive analysis, 162 distinct gastrointestinal AE PTs demonstrated a statistically significant disproportionality signal (ROR_025_ > 1) with vedolizumab exposure (Supplementary Table S3). Focusing on signals with at least 20 reported cases to enhance robustness, 95 such PTs were identified. Figure 2 presents the ROR and 95% CI for these 95 significant gastrointestinal AE signals, ordered by the number of reports. The most frequently reported signals included diarrhoea (n = 5,420, ROR = 2.25), abdominal pain (n = 4,770, ROR = 6.16), haematochezia (n = 3,855, ROR = 20.83), frequent bowel movements (n = 3,052, ROR = 35.05), and rectal haemorrhage (n = 1,346, ROR = 9.38). Signals exhibiting the strongest disproportionality (highest ROR_025_ values) included mucous stools (ROR_025_ = 57.70), defaecation urgency (ROR_025_ = 39.81), frequent bowel movements (ROR_025_ = 33.71), pouchitis (ROR_025_ = 24.79), and intestinal stenosis (ROR_025_ = 21.53). Approximately 70 of the 95 significant gastrointestinal AE PTs with ≥20 reports are not explicitly listed as common AEs in the current vedolizumab drug label.

### 3.3 Serious vs. non-serious cases

Analysis of serious versus non-serious gastrointestinal AE reports revealed significant differences in demographic characteristics. As shown in Table 2, there were statistically significant differences in gender (*χ* ^2^ = 9.65, *p* = 0.002), body weight (*χ* ^2^ = 17.38, *p* < 0.001), and age distribution (*χ* ^2^ = 78.50, *p* < 0.001) between severe and non-severe cases. Reports classified as serious were significantly more likely to involve male patients and patients with lower body weight among reports with available data. Regarding age, the proportion of serious reports was highest in the 18∼45 age group (96.12%) and 46∼65 age group (95.47%), compared to the pediatric (<18 years, 91.43%) and elderly (>65 years, 92.00%) populations. Table 2 details the distribution of the top 10 most frequent AE types, while the full comparison for all significant AEs is provided in Supplementary Table S4. Thirty-three specific PTs, such as diarrhoea, nausea, haematochezia, vomiting, and constipation, were significantly more likely to be reported as serious events (*p* < 0.05 for each).

**TABLE 2:** Differences in clinical characteristics of serious and non-serious reports.

Characteristics	Non-serious cases (N = 1,779)	Serious cases (N = 16,164)	Statistic (*χ* ^2^)	*p* value
Gender, n (%)				
Female	1,026 (9.96)	9,280 (90.04)	9.65	0.002^a^
Male	621 (8.56)	6,637 (91.44)	​	​
Weight (kg), n (%)				
<50	32 (12.70)	220 (87.30)	17.38	<0.001^a^
50∼100	274 (17.06)	1,332 (82.94)	​	​
>100	44 (28.57)	110 (72.43)	​	​
Age (years), n (%)				
<18	27 (8.57)	288 (91.43)	78.50	<0.001^a^
18∼45	214 (3.88)	5,303 (96.12)	​	​
46∼65	197 (4.53)	4,151 (95.47)	​	​
>65	236 (8.00)	2,714 (92.00)	​	​
Types of AEs, n (%)				
Diarrhoea	490 (9.04)	4,930 (90.96)	6.62^c^	<0.05^a^
Abdominal pain	387 (8.11)	4,383 (91.89)	0^c^	0.28^a^
Haematochezia*	227 (5.89)	3,628 (94.11)	28.29^c^	<0.05^a^
Frequent bowel movements*	213 (6.98)	2,839 (93.02)	5.73^c^	0.13^a^
Nausea	345 (14.99)	1,957 (85.01)	151.57^c^	<0.05^a^
Vomiting	160 (10.26)	1,399 (89.74)	9.48^c^	<0.05^a^
Rectal haemorrhage	75 (5.57)	1,271 (94.43)	11.88^c^	<0.05^a^
Constipation	125 (9.38)	1,207 (90.62)	2.69^c^	0.02^a^
Abdominal pain upper	110 (9.08)	1,102 (90.92)	1.34^c^	0.08^a^
Intestinal obstruction	42 (4.08)	987 (95.92)	22.64^c^	<0.05^a^

Only the top 10 most frequently reported AEs, are listed below. The full list of comparisons for all significant AEs, is provided in Supplementary Table S4.

^a^Proportions were compared using Pearson’s chi-squared (*χ* ^2^) test; ^b^ Proportions were compared using Fisher’s exact test; ^c^ The *χ* ^2^ statistic of the Pearson’s chi-squared (*χ* ^2^) test; ^d^ The Fisher’s exact statistic of the Fisher’s exact test. * Emerging findings of vedolizumab-associated gastrointestinal AEs, from FAERS, database.

Abbreviation: AEs, adverse events; n, number of cases.

### 3.4 Stratification analysis

**FIGURE 3:**
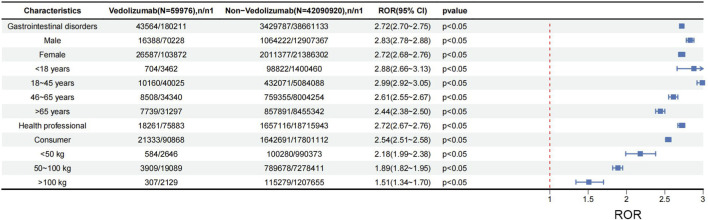
The stratification analysis of vedolizumab-related gastrointestinal disorders. Abbreviations: n, number of cases of total AEs associated with the given drug; n1, number of cases without suspected AEs (i.e., total AEs excluding suspected ones) associated with the given drug; ROR, reporting odds ratio; CI, confidence interval.

Figure 3 illustrates the results of the stratification analysis for the overall SOC “Gastrointestinal disorders.” It indicates that different subgroups (such as gender, age, weight, and reporter type) have a strong statistical association with the occurrence of vedolizumab-related gastrointestinal disease. In gender stratification, ROR was 2.83 for males and 2.72 for females, suggesting a significant difference both in males and females. In the age stratification, the 18∼45 age group exhibited the most prominent disproportionality signal (ROR = 2.99), closely followed by the 46∼65 age group. This indicates that reports involving adult patients constituted a prominent proportion of vedolizumab-related gastrointestinal AEs, although this may reflect usage patterns. In the weight stratification, there were significant differences among different body weights, and the ROR value in the <50 kg group was 2.18. After grouping by reporter type, it could be seen that there is a more significant difference ROR 2.72 in the reports of healthcare professionals.

### 3.5 Clinical relevance assessment of disproportionate signals

Based on the analysis of the 95 significant gastrointestinal AE PTs with ≥20 reports, 33 (35.11%) were identified as IMEs according to EMA criteria, with intestinal perforation being a DME. Applying our semi-quantitative signal strength assessment system (Supplementary Table S2), the 95 signals were classified into relevance levels. Table 3 presents the 35 PTs (36.84%) classified as moderate relevance (score 3∼5), while the 60 PTs (63.16%) classified as low relevance (score 0∼2) are provided in Supplementary Table S5. No signals reached the high relevance threshold (score 6∼8) in this analysis. Signals with the highest scores in the moderate category (score 4) included haematochezia (n = 3,855, ROR_025_ = 20.14) and intestinal perforation (n = 131, ROR_025_ = 2.92). Other notable moderate priority signals (score 3) included diarrhoea (n = 5,420, ROR_025_ = 2.19), abdominal pain (n = 4,770, ROR_025_ = 5.98), frequent bowel movements (n = 3,052, ROR_025_ = 33.71), rectal haemorrhage (n = 1,346, ROR_025_ = 8.88), and intestinal stenosis (n = 334, ROR_025_ = 21.53). As previously noted, approximately 70 of these 95 PTs with significant RORs, such as frequent bowel movements (n = 3,052, ROR_025_ = 33.71) and defaecation urgency (n = 923, ROR_025_ = 39.81), are not explicitly listed as common AEs in the current vedolizumab drug label.

**TABLE 3:** Clinical relevance assessment results (moderate relevance signals).

PTs	N	ROR_025_	Death (n)	IMEs/DMEs	Relevance level (score)
Diarrhoea	5,420	2.19	55	NA	Moderate (3)
Abdominal pain	4,770	5.98	26	NA	Moderate (3)
Haematochezia*	3,855	20.14	7	IME	Moderate (4)
Frequent bowel movements*	3,052	33.71	15	NA	Moderate (3)
Rectal haemorrhage	1,346	8.88	5	IME	Moderate (3)
Intestinal obstruction	1,029	7.52	25	IME	Moderate (3)
Colitis*	491	3.28	8	IME	Moderate (3)
Intestinal stenosis	334	21.53	2	IME	Moderate (3)
Diarrhoea haemorrhagic*	306	8.59	4	IME	Moderate (3)
Small intestinal obstruction	259	5.67	6	IME	Moderate (3)
Inflammatory bowel disease*	176	7.24	0	IME	Moderate (3)
Large intestinal stenosis*	160	18.57	2	IME	Moderate (3)
Intestinal perforation*	131	2.92	11	DME	Moderate (4)
Ileal stenosis	120	18.18	1	IME	Moderate (3)
Large intestinal ulcer*	86	8.12	0	IME	Moderate (3)
Colitis microscopic*	83	3.56	0	IME	Moderate (3)
Intestinal fistula*	76	12.96	1	IME	Moderate (3)
Intestinal haemorrhage*	75	3.11	3	IME	Moderate (3)
Intestinal ulcer*	61	9.01	2	IME	Moderate (3)
Gastrointestinal obstruction*	60	5.14	0	IME	Moderate (3)
Large intestine perforation	53	2.02	3	IME	Moderate (3)
Small intestinal stenosis	50	10.22	0	IME	Moderate (3)
Ileal ulcer*	43	9.20	0	IME	Moderate (3)
Proctitis ulcerative*	36	17.46	1	IME	Moderate (3)
Colon dysplasia	32	20.93	1	IME	Moderate (3)
Enterocutaneous fistula*	31	7.81	0	IME	Moderate (3)
Anal stenosis*	30	12.64	0	IME	Moderate (3)
Gastrointestinal fistula*	26	5.29	0	IME	Moderate (3)
Volvulus*	25	3.99	0	IME	Moderate (3)
Rectal stenosis*	25	1.97	0	IME	Moderate (3)
Enterovesical fistula	23	6.63	1	IME	Moderate (3)
Large intestinal haemorrhage*	23	1.87	0	IME	Moderate (3)
Rectal ulcer*	23	3.91	0	IME	Moderate (3)
Small intestinal perforation*	21	2.21	3	IME	Moderate (3)
Immune-mediated enterocolitis*	20	1.02	0	IME	Moderate (3)

This table displays signals classified as “Moderate Relevance” (Score 3∼5). Signals classified as “Low Relevance” (Score 0∼2) are presented in Supplementary Table S5.

*New and unexpected signals, not previously reported in the drug label, emerging findings from FAERS, database.

Abbreviations: PTs, preferred terms; N, the total number of reported cases for a given PT; ROR_025_, the lower limit of 95% confidence interval of ROR; IMEs, important medical events; DMEs, designated medical events; NA, not applicable (for relevant criteria).

### 3.6 Time-to-onset analysis

TTO data were available for a substantial subset of the reports. Table 4 summarizes the TTO distribution for signals categorized by clinical priority. The median TTO for gastrointestinal AEs classified as moderate priority (n = 8,773 reports with TTO data) was 190 days (IQR 77∼435 days). For low priority signals (n = 6,450 reports with TTO data), the median TTO was also at 190 days (IQR 78∼462 days). The WSP analysis for both moderate (β = 0.83, 95% CI = 0.81∼0.84) and low priority (β = 0.82, 95% CI = 0.81∼0.84) signals yielded β values significantly less than 1. This indicates an “early failure” pattern for the occurrence of these gastrointestinal AEs, suggesting that the hazard of occurrence is highest relatively early in the treatment course and decreases over time.

**TABLE 4:** Time-to-onset analysis for signals with moderate/weak prioritization.

Prioritization	Cases (n)	TTO (days)		Weibull distribution				Failure type
				Scale parameter		Shape parameter		
		Median (IQR)	Min ∼ max	α	95% CI	β	95% CI	
Moderate	8,773	190 (77∼435)	1∼7,558	313.65	305.30∼322.00	0.83	0.81∼0.84	Early failure
Low	6,450	190 (78∼462)	1∼7,558	323.89	313.78∼334.01	0.82	0.81∼0.84	Early failure

Abbreviations: n, number of cases with available time-to-onset; TTO, time-to-onset; IQR, interquartile range; α, scale parameter; β, shape parameter.

## 4 Discussion

The present pharmacovigilance study provides a comprehensive characterization of vedolizumab-associated gastrointestinal AEs using extensive real-world data from the FAERS. Our disproportionality analysis, encompassing over a decade of post-marketing surveillance, has identified both well-established and potential novel gastrointestinal safety signals, offering critical insights into the evolving gastrointestinal toxicity profile of this widely utilized gut-selective biologic. These findings are particularly relevant given the expanding use of vedolizumab, including newer formulations, its increasing use in diverse patient populations, and the prolonged exposure observed in routine clinical practice. The significant increase in the absolute number of gastrointestinal AE reports for vedolizumab from 2021 onwards (Table 1) likely reflects this broader adoption, including the introduction and uptake of subcutaneous formulations, and an expanding patient base (Colombel et al., 2017; Sandborn et al., 2020).

### 4.1 Comparison of safety signals between different studies

Our study included 17,943 reports of vedolizumab-associated gastrointestinal AEs after rigorous preprocessing. To mitigate confounding by indication—a significant challenge in pharmacovigilance for chronic diseases—we explicitly excluded AE terms referring to underlying IBD diagnoses (e.g., “ulcerative colitis,” “Crohn’s disease,” “disease progression”) (Xu et al., 2025). Despite this refinement, distinguishing drug-induced symptoms from those inherent to IBD pathophysiology (such as bleeding or changes in bowel habits) remains inherently difficult in spontaneous reporting data, even with careful exclusion of diagnostic terms. While a sensitivity analysis comparing results with and without excluding diagnostic terms could assess the robustness of these signals, it cannot definitively differentiate drug toxicity from disease activity. Ideally, comparing patients with IBD-related indications versus non-IBD indications would clarify this; however, since vedolizumab is prescribed almost exclusively for IBD, such a control group is not feasible within FAERS. Therefore, the elevated RORs observed in our study likely represent a composite of true drug-induced AEs, paradoxical reactions, and residual underlying disease activity. These findings should therefore be interpreted as strong statistical associations warranting clinical monitoring rather than definitive evidence of causality.

The most frequently reported gastrointestinal events in our analysis—diarrhoea (n = 5,420), abdominal pain (n = 4,770), constipation (n = 1,332), and abdominal distension (n = 1,077)—are broadly consistent with the gastrointestinal AE profiles observed in vedolizumab’s pivotal clinical trials, such as GEMINI I & II and subsequent analyses (Colombel et al., 2017; Sands et al., 2019). These common events, though potentially overlapping with IBD symptoms, showed significant disproportionality signals, suggesting a potential drug association or exacerbation. However, our real-world data also highlighted remarkably high reporting frequencies and strong disproportionality signals for events such as haematochezia (n = 3,855, ROR 20.83), frequent bowel movements (n = 3,052, ROR 35.05), and rectal haemorrhage (n = 1,346, ROR 9.38). These findings, corroborated by a recent network meta-analysis by Macaluso et al. which found vedolizumab associated with a higher risk of certain gastrointestinal AEs compared to placebo (Macaluso et al., 2023), underscore the general observation of gastrointestinal AEs and our study’s contribution of more granular data on the spectrum and relative reporting frequency of specific events.

Importantly, our results are further supported by a recent retrospective cohort study by Milašinović et al., which also investigated the real-world safety of vedolizumab in IBD patients using FAERS signal analysis (Milašinović et al., 2025). This independent analysis reported a similar spectrum of AEs, particularly highlighting gastrointestinal disorders, infections, and general disorders, consistent with our findings. The congruence across these FAERS-based pharmacovigilance studies, especially regarding the prominence of gastrointestinal events like abdominal pain and nausea, reinforces the reliability of our methodology in identifying drug-event associations and strengthens the validity of the observed safety signals for vedolizumab in a real-world setting. This corroboration underscores the utility of spontaneous reporting systems like FAERS in providing complementary and valuable insights into the post-marketing safety profile of biological therapies.

The clinical interpretation of these overlapping symptoms is multifaceted. A patient experiencing worsened diarrhea or abdominal pain while on vedolizumab presents a fundamental diagnostic challenge: this could reflect primary non-response or secondary loss of response (indicating the drug is failing to suppress the disease), or conversely, a true drug-induced adverse event (where vedolizumab itself causes paradoxical inflammation or hypersensitivity). Furthermore, the drug might increase susceptibility to gastrointestinal infections (like Clostridioides difficile), whose symptoms closely mimic an IBD flare. Since FAERS lacks the granular clinical detail necessary to definitively differentiate these scenarios (e.g., baseline disease severity, endoscopic findings, or inflammatory biomarkers), a high ROR for an IBD-like symptom does not definitively establish causality. Instead, it signals a need for clinicians to meticulously evaluate such events, considering the full clinical picture given vedolizumab’s targeted mechanism of action within the gut.

From a mechanistic perspective, vedolizumab blocks α4β7 integrin-mediated lymphocyte homing to the gut mucosa (Feagan et al., 2013; Soler et al., 2009). While intended to reduce inflammation, this targeted immunosuppression could theoretically alter local gut immune homeostasis, affect mucosal barrier integrity, or influence enteric nervous system function. For instance, for signals like intestinal perforation or bleeding, while they can be complications of severe IBD, it is plausible that vedolizumab, by modulating lymphocyte trafficking and potentially altering mucosal healing processes, might contribute to or exacerbate such events in susceptible patients. Some evidence suggests that, rarely, biologics can lead to paradoxical inflammatory or motility disorders, or impair mucosal healing, potentially contributing to events like bleeding, perforation, or changes in bowel habits observed in our analysis (Zhan et al., 2025). Unlike previous broad overviews, our study specifically delineates this granular spectrum of toxicity. We identified 95 significant gastrointestinal AE PTs with at least 20 reports, and notably, approximately 70 of these (e.g., “defaecation urgency,” “mucous stools”) are not explicitly listed as common AEs in current vedolizumab prescribing information. While these may relate to disease activity, their strong disproportionality signals warrant clinical attention as potential indicators of suboptimal treatment control or specific drug effects, highlighting the unique value of this pharmacovigilance analysis.

### 4.2 Serious vs. non-serious reports

Clinical trials typically report a low frequency of serious AEs, with most gastrointestinal events being mild-to-moderate and infrequently leading to discontinuation (Colombel et al., 2017; Sandborn et al., 2020; Sands et al., 2019). In contrast, our real-world FAERS analysis revealed that 90.09% of vedolizumab-associated gastrointestinal AE reports were classified as serious. This high proportion is consistent with pharmacovigilance data for other biologics used in IBD, such as anti-TNF agents (infliximab, adalimumab), where serious reporting rates in FAERS often exceed 85% (Joshi et al., 2025). This discrepancy primarily reflects the inherent “notoriety bias” of spontaneous reporting systems, where healthcare professionals and consumers are significantly more likely to report severe events requiring hospitalization than mild symptoms.

Despite this inherent bias, our data (Table 2) identified specific factors associated with reports being classified as serious. Statistically significant differences were observed across gender, weight, and age groups. Notably, regarding age, our data indicated a higher proportion of serious outcomes in adult patients (18∼65 years) compared to the elderly (>65 years). This finding contrasts with general clinical observations where elderly patients typically exhibit higher frailty and risk of severe AEs (Kirchgesner et al., 2018). However, this specific association must be interpreted with caution due to a substantial “missing data bias” identified in our dataset: demographic information (specifically age) was missing in over 60% of “non-serious” reports, whereas serious reports had much higher completeness. This differential missingness likely artificially inflates the severity rate in the adult population. Therefore, the observed age-related severity pattern may reflect reporting dynamics rather than a true biological susceptibility.

Regarding gender and weight, while female patients generally submit more AE reports overall (aligning with IBD epidemiological patterns) (Salem et al., 2024), in our cohort, the finding that serious gastrointestinal events were more frequently reported among males warrants further attention. Similarly, the association between lower body weight and higher severity could reflect pharmacokinetic differences, disease severity, or confounding by other factors. As expected, clinically critical events like rectal haemorrhage and intestinal obstruction were significantly more likely to be coded as serious. These observations require validation in prospective studies to distinguish true biological predispositions from reporting influences or underlying patient characteristics not fully captured in FAERS.

### 4.3 Clinical relevance assessment of disproportionate signals

Leveraging a semi-quantitative assessment system allowed us to prioritize the detected signals based on their potential clinical impact, thereby aiming to provide potential insights for clinicians and enhance focused pharmacovigilance efforts. Consistent with our results section (Table 3), this analysis classified 35 signals as moderate relevance (score 3∼5) and 60 as low relevance (score 0∼2), with no signals reaching the highest relevance threshold (score 6∼8) based on our defined criteria. Among the moderate relevance signals, haematochezia (score 4, n = 3,855, IME) and intestinal perforation (score 4, n = 131, DME) are clinically particularly concerning due to their nature and potential severity. Other moderate relevance signals (score 3) included rectal haemorrhage, haemorrhagic diarrhoea, intestinal haemorrhage, and frequent bowel movements. The strong disproportionality signals for haematochezia (ROR_025_ = 20.14) and frequent bowel movements (ROR_025_ = 33.71) further underscore their clinical significance despite their moderate relevance score based on the composite criteria. The ROR_025_ value for intestinal perforation is also high (2.92), and classifying it as moderate (score 4) is consistent with its inclusion as a designated medical event (DME). These findings are biologically plausible given vedolizumab’s mechanism, which, by altering lymphocyte trafficking, could potentially impair mucosal healing or host defense mechanisms, theoretically predisposing patients to bleeding or perforation, especially in the context of pre-existing mucosal damage in IBD (Feagan et al., 2013; Soler et al., 2009).

The substantial proportion of signals classified as low relevance (63.16%) indicates that the majority of disproportional gastrointestinal AEs associated with vedolizumab are likely less severe or more manageable in clinical practice. Nevertheless, vigilance is required for all disproportionately reported events, especially those considered IMEs or DMEs, regardless of their final relevance score in this system. It is critical to reiterate that this scoring system is a heuristic and its “priority levels” should not be directly equated with definitive clinical significance due to its *ad hoc* weighting and lack of external validation. Our findings highlight the necessity for enhanced caution in post-marketing surveillance, particularly for less commonly acknowledged events such as defaecation urgency and mucous stools, which showed strong signals but were classified as low relevance based on the composite score (Table 3), potentially warranting re-evaluation or closer monitoring in specific contexts.

### 4.4 Time-to-onset analysis

The TTO analysis (Table 4) revealed a median TTO of approximately 190 days for both moderate and low priority gastrointestinal AE signals, with Weibull shape parameters (β) significantly less than 1. This indicates an “early failure” temporal pattern, suggesting that the risk or reporting frequency of these vedolizumab-associated gastrointestinal AEs is highest relatively early in the treatment course and tends to decrease over time. This temporal profile contrasts with the delayed onset often observed with some systemic immune-mediated adverse events associated with broader immunosuppressants or immune checkpoint inhibitors (Ramos-Casals et al., 2020). This distinct temporal pattern for gastrointestinal AEs provides valuable information for clinicians regarding the monitoring window after initiating vedolizumab therapy.

### 4.5 Confounding by concomitant medications

Another important limitation inherent to spontaneous reporting systems is the inability to comprehensively account for the confounding effects of concomitant medications. Patients with IBD often receive multiple therapies, including other immunosuppressants, corticosteroids, or antibiotics, which could also contribute to gastrointestinal AEs. The FAERS database, while invaluable for signal detection, lacks the granular clinical detail, such as complete concomitant medication histories, dosages, and precise timelines, necessary to definitively disentangle the contribution of vedolizumab from other co-administered agents. Therefore, our findings reflect associations with vedolizumab in a real-world setting where polypharmacy is common, but cannot definitively exclude the contribution of other therapies to the observed AEs.

## 5 Conclusion

This comprehensive real-world pharmacovigilance analysis of the FAERS database significantly enhances our understanding of the gastrointestinal safety profile of vedolizumab. It is crucial to emphasize that while this study identifies statistical associations, it does not establish causality, which is an inherent limitation of observational pharmacovigilance data. It identifies previously recognized gastrointestinal AEs and suggests a substantial number of potentially underrecognized associations that warrant further investigation. While overall, gastrointestinal AEs associated with vedolizumab are often manageable, this study highlights the importance of clinical vigilance, particularly regarding serious events such as intestinal perforation, severe bleeding (e.g., haematochezia, rectal haemorrhage), and potential motility disorders. The findings suggest that male patients and those with lower body weight may be at higher risk for serious gastrointestinal AEs, warranting closer monitoring in these subgroups. Furthermore, the “early failure” TTO pattern suggests that enhanced vigilance is particularly crucial during the initial months of vedolizumab therapy. The potentially underrecognized signals identified warrant further investigation to clarify their association with vedolizumab and inform updated prescribing information. Despite the inherent limitations of spontaneous reporting data, this study provides critical real-world safety information that complements clinical trial data and can guide clinical practice to optimize patient care and safety during vedolizumab treatment. These findings are hypothesis-generating and require validation through prospective observational studies or clinical trials before they can influence clinical practice guidelines.

## Data Availability

Publicly available datasets were analyzed in this study. This data can be found here: The dataset used in this study is available from an online repository: the FAERS Public Dashboard (https://www.fda.gov/drugs/questions-and-answers-fdas-adverse-event-reporting-system-faers/fda-adverse-event-reporting-system-faers-public-dashboard).

## References

[B1] Abu-Sbeih H. Ali F. S. Alsaadi D. Jennings J. Luo W. Gong Z. (2018). Outcomes of vedolizumab therapy in patients with immune checkpoint inhibitor-induced colitis: a multi-center study. J. Immunother. Cancer 6, 142. 10.1186/s40425-018-0461-4 30518410 PMC6280383

[B2] Ahuja D. Murad M. H. Ma C. Jairath V. Singh S. (2023). Comparative speed of early symptomatic remission with advanced therapies for moderate-to-severe ulcerative colitis: a systematic review and network meta-analysis. Am. J. Gastroenterol. 118, 1618–1625. 10.14309/ajg.0000000000002263 36976548

[B3] Caster O. Aoki Y. Gattepaille L. M. Grundmark B. (2020). Disproportionality analysis for pharmacovigilance signal detection in small databases or subsets: recommendations for limiting false-positive associations. Drug Saf. 43, 479–487. 10.1007/s40264-020-00911-w 32008183 PMC7165139

[B4] Cecco S. Puligheddu S. Fusaroli M. Gerratana L. Yan M. Zamagni C. (2024). Emerging toxicities of antibody-drug conjugates for breast cancer: clinical prioritization of adverse events from the FDA adverse event reporting system. Target Oncol. 19, 435–445. 10.1007/s11523-024-01058-9 38696126 PMC11111510

[B5] Chen C. Wu B. Zhang C. Xu T. (2021). Immune-related adverse events associated with immune checkpoint inhibitors: an updated comprehensive disproportionality analysis of the FDA adverse event reporting system. Int. Immunopharmacol. 95, 107498. 10.1016/j.intimp.2021.107498 33725634

[B6] Cohen R. D. Bhayat F. Blake A. Travis S. (2020). The safety profile of vedolizumab in ulcerative colitis and Crohn’s disease: 4 years of global post-marketing data. J. Crohns Colitis 14, 192–204. 10.1093/ecco-jcc/jjz137 31504340

[B7] Colombel J. F. Sands B. E. Rutgeerts P. Sandborn W. Danese S. D’Haens G. (2017). The safety of vedolizumab for ulcerative colitis and Crohn’s disease. Gut 66, 839–851. 10.1136/gutjnl-2015-311079 26893500 PMC5531223

[B8] Feagan B. G. Rutgeerts P. Sands B. E. Hanauer S. Colombel J. F. Sandborn W. J. (2013). Vedolizumab as induction and maintenance therapy for ulcerative colitis. N. Engl. J. Med. 369, 699–710. 10.1056/NEJMoa1215734 23964932

[B9] Gastaldon C. Schoretsanitis G. Arzenton E. Raschi E. Papola D. Ostuzzi G. (2022). Withdrawal syndrome following discontinuation of 28 antidepressants: pharmacovigilance analysis of 31,688 reports from the WHO spontaneous reporting database. Drug Saf. 45, 1539–1549. 10.1007/s40264-022-01246-4 36400895 PMC9676852

[B10] Guo H. Wang B. Yuan S. Wu S. Liu J. He M. (2022). Corrigendum: neurological adverse events associated with esketamine: a disproportionality analysis for signal detection leveraging the FDA adverse event reporting system. Front. Pharmacol. 13, 1075966. 10.3389/fphar.2022.1075966 36438806 PMC9683689

[B11] Joshi A. Gawey L. Saadi C. Naidoo P. Rick J. W. Park S. Y. (2025). Postmarketing safety surveillance of adalimumab, secukinumab, and infliximab in hidradenitis suppurativa: an analysis of the FDA adverse events reporting system (FAERS) database. J. Am. Acad. Dermatol. 92, 1434–1435. 10.1016/j.jaad.2025.02.031 39978679

[B12] Kirchgesner J. Lemaitre M. Carrat F. Zureik M. Carbonnel F. Dray-Spira R. (2018). Risk of serious and opportunistic infections associated with treatment of inflammatory bowel diseases. Gastroenterology 155, 337–346. 10.1053/j.gastro.2018.04.012 29655835

[B13] Macaluso F. S. Ventimiglia M. Orlando A. (2023). Effectiveness and safety of vedolizumab in inflammatory bowel disease: a comprehensive meta-analysis of observational studies. J. Crohns Colitis 17, 1217–1227. 10.1093/ecco-jcc/jjad043 36913311

[B14] Mao W. Jiang J. Xia Y. Zhang L. (2025). Analysis of postmarketing neuropsychiatric adverse events of avapritinib based on the FDA adverse event reporting system. Sci. Rep. 15, 3108. 10.1038/s41598-025-86959-z 39856211 PMC11760955

[B15] Milašinović B. Vezmar Kovačević S. Marković S. Jovanović M. Knežević Ivanovski T. Kralj Đ. (2025). Real-world safety of vedolizumab in inflammatory bowel disease: a retrospective cohort study supported by FAERS signal analysis. Pharmaceuticals (Basel) 18, 1127. 10.3390/ph18081127 40872519 PMC12389730

[B16] Morris R. Ali R. Cheng F. (2024). Drug repurposing using FDA adverse event reporting system (FAERS) database. Curr. Drug Targets 25, 454–464. 10.2174/0113894501290296240327081624 38566381

[B17] Osei S. P. Akomaning E. Florut T. F. Sodhi M. Lacy B. E. Aldhaleei W. A. (2024). Gastrointestinal safety assessment of GLP-1 receptor agonists in the US: a real-world adverse events analysis from the FAERS database. Diagnostics 14, 2829. 10.3390/diagnostics14242829 39767190 PMC11675942

[B18] Ramos-Casals M. Brahmer J. R. Callahan M. K. Flores-Chávez A. Keegan N. Khamashta M. A. (2020). Immune-related adverse events of checkpoint inhibitors. Nat. Rev. Dis. Prim. 6, 38. 10.1038/s41572-020-0160-6 32382051 PMC9728094

[B19] Ren G. Huang P. Ding Y. Ma X. (2025). Risk of drug-induced pericardial effusion: a disproportionality analysis of the FAERS database. BMC Pharmacol. Toxicol. 26, 27. 10.1186/s40360-025-00867-6 39920868 PMC11806542

[B20] Salem D. A. El-Ijla R. AbuMusameh R. R. Zakout K. A. Abu Halima A. Y. Abudiab M. T. (2024). Sex-related differences in profiles and clinical outcomes of inflammatory bowel disease: a systematic review and meta-analysis. BMC Gastroenterol. 24, 425. 10.1186/s12876-024-03514-2 39580396 PMC11585250

[B21] Sandborn W. J. Feagan B. G. Rutgeerts P. Hanauer S. Colombel J. F. Sands B. E. (2013). Vedolizumab as induction and maintenance therapy for Crohn’s disease. N. Engl. J. Med. 369, 711–721. 10.1056/NEJMoa1215739 23964933

[B22] Sandborn W. J. Baert F. Danese S. Krznarić Ž. Kobayashi T. Yao X. (2020). Efficacy and safety of vedolizumab subcutaneous formulation in a randomized trial of patients with ulcerative colitis. Gastroenterology 158, 562–572. 10.1053/j.gastro.2019.08.027 31470005

[B23] Sands B. E. Van Assche G. Tudor D. Akhundova-Unadkat G. Curtis R. I. Tan T. (2019). Vedolizumab in combination with corticosteroids for induction therapy in Crohn’s disease: a post hoc analysis of GEMINI 2 and 3. Inflamm. Bowel Dis. 25, 1375–1382. 10.1093/ibd/izy384 30615117 PMC6635819

[B24] Shu Y. Ding Y. Dai B. Zhang Q. (2022). A real-world pharmacovigilance study of axitinib: data mining of the public version of FDA adverse event reporting system. Expert Opin. Drug Saf. 21, 563–572. 10.1080/14740338.2022.2016696 34918584

[B25] Shu Y. He X. Wu P. Liu Y. Ding Y. Zhang Q. (2022). Gastrointestinal adverse events associated with semaglutide: a pharmacovigilance study based on FDA adverse event reporting system. Front. Public Health 10, 996179. 10.3389/fpubh.2022.996179 36339230 PMC9631444

[B26] Soler D. Chapman T. Yang L. L. Wyant T. Egan R. Fedyk E. R. (2009). The binding specificity and selective antagonism of vedolizumab, an anti-alpha4beta7 integrin therapeutic antibody in development for inflammatory bowel diseases. J. Pharmacol. Exp. Ther. 330, 864–875. 10.1124/jpet.109.153973 19509315

[B27] Vestergaard Kvist A. Faruque J. Vallejo-Yagüe E. Weiler S. Winter E. M. Burden A. M. (2021). Cardiovascular safety profile of romosozumab: a pharmacovigilance analysis of the US food and drug administration adverse event reporting system (FAERS). J. Clin. Med. 10, 1660. 10.3390/jcm10081660 33924496 PMC8070537

[B28] Xu Q. Zhang J. Tang W. Zhou M. Zhang X. Yuan P. (2025). Data mining and analysis of adverse events of vedolizumab based on the FAERS database. Sci. Rep. 15, 278. 10.1038/s41598-024-75421-1 39747183 PMC11696444

[B29] Yang C. Zhao W. Chen H. Yao Y. Zhang J. (2024). Cardiac adverse events associated with lacosamide: a disproportionality analysis of the FAERS database. Sci. Rep. 14, 16202. 10.1038/s41598-024-67209-0 39003359 PMC11246456

[B30] Zhan Z. Q. Li J. X. Chen Y. X. Fang J. Y. (2025). Association between biologics and Janus kinase inhibitors with inflammatory bowel disease as paradoxical reactions: a real-world assessment. United Eur. Gastroenterol. J. 13, 531–541. 10.1002/ueg2.12719 39676727 PMC12090828

[B31] Zhou C. Peng S. Lin A. Jiang A. Peng Y. Gu T. (2023). Psychiatric disorders associated with immune checkpoint inhibitors: a pharmacovigilance analysis of the FDA adverse event reporting system (FAERS) database. EClinicalMedicine 59, 101967. 10.1016/j.eclinm.2023.101967 37131541 PMC10149185

[B32] Zou S. Ouyang M. Zhao Y. Cheng Q. Shi X. Sun M. (2024). A disproportionality analysis of adverse events caused by GnRHas from the FAERS and JADER databases. Front. Pharmacol. 15, 1392914. 10.3389/fphar.2024.1392914 39027335 PMC11254796

