# Supplementary material for: Beyond the label: real-world gastrointestinal adverse events associated with vedolizumab – a decade of FAERS pharmacovigilance
Source: Front Pharmacol. 2026 Jan 14;16:1700820. doi: 10.3389/fphar.2025.1700820 (PMC12847282; doi:10.3389/fphar.2025.1700820)
Supplement: Supplementary file 1 [file Supplementaryfile1.docx]

**Supplementary Information for**

**Beyond the label: real-world gastrointestinal adverse events associated with vedolizumab – A decade of FAERS pharmacovigilance**

**Supplementary Table S1**

Calculation of reporting odds ratio (ROR) and its 95% confidence interval (95% CI).

|  | Reports with target AEs | Reports without target AEs |
| --- | --- | --- |
| Reports with vedolizumab | a | b |
| Reports without vedolizumab | c | d |

**Note:** The calculation formulas are shown below:

1. ROR = ad/b/c
2. 95% CI = e^ln(ROR) ± 1.96(1/a+1/b+1/c+1/d)^0.5^

**Abbreviation:** AEs, adverse events; ROR, reporting odds ratio; CI, confidence interval. a, number of reports containing both the target drug (vedolizumab) and target AEs; b, number of reports containing other AEs of the target drug; c, number of reports containing the target AEs of other drugs; d, number of reports containing other drugs and other AEs.

**Supplementary Table S2**

Semi-quantitative signal strength assessment system prioritizing the statistically significant disproportionality signals based on their potential clinical impact.

| Assessment items | 2 points | 1 point | 0 point |
| --- | --- | --- | --- |
| Reporting frequency (cases/non-cases) | ≥10% | 1~10% | <1% |
| Signal stability (consistency across disproportionality analyses) | 3 of 3 | 2 of 3 | 1 of 3 |
| Reported case fatality rate (proportion of reports with death as outcome) | >50% | 25~50% | <25% |
| Clinical relevance (serious likely drug-attributable AEs) | DME | IME | None |

**Note:** Reporting frequency: the proportion of the AE of interest as compared with other AEs (i.e., the ratio between cases and non-cases). To mirror clinical trials, the following traditional categories were used: very common (≥10%), common (1~10%), and uncommon (<1%).

Signal stability: consistency/robustness of disproportionality signals across multiple analyses. Maximum score was awarded to full consistency (disproportionality signals in 3 out of 3 analyses on the basis of diferent comparators).

Reported case fatality rate: the proportion of reports where death was recorded, as compared with all AEs.

IMEs and DMEs are developed and updated by EMA (European Medicines Agency, 2020). AEs, adverse events; DMEs, designated medical events; IMEs, important medical events.

**Supplementary Table S3**

Results of gastrointestinal AE PTs with a statistically significant disproportionality signal (ROR_025_ > 1) with vedolizumab exposure. Criteria: n ≥ 3.

| Preferred terms (PTs) | Number of cases (n) | ROR (95% CI) |
| --- | --- | --- |
| Diarrhoea | 5,420 | 2.25 (2.19-2.31) |
| Abdominal pain | 4,770 | 6.16 (5.98-6.34) |
| Haematochezia | 3,855 | 20.83 (20.14-21.54) |
| Frequent bowel movements | 3,052 | 35.05 (33.71-36.44) |
| Rectal haemorrhage | 1,346 | 9.38 (8.88-9.91) |
| Constipation | 1,332 | 1.69 (1.60-1.78) |
| Abdominal pain upper | 1,212 | 1.65 (1.56-1.75) |
| Abdominal distension | 1,077 | 2.96 (2.78-3.14) |
| Intestinal obstruction | 1,029 | 8.00 (7.52-8.52) |
| Defaecation urgency | 923 | 42.77 (39.81-45.94) |
| Mucous stools | 910 | 62.20 (57.7-67.04) |
| Abdominal discomfort | 774 | 1.16 (1.08-1.25) |
| Flatulence | 689 | 3.60 (3.34-3.88) |
| Faeces soft | 552 | 19.01 (17.41-20.75) |
| Colitis | 491 | 3.59 (3.28-3.92) |
| Haemorrhoids | 397 | 6.29 (5.69-6.95) |
| Gastrointestinal inflammation | 395 | 13.61 (12.29-15.08) |
| Abdominal pain lower | 391 | 4.56 (4.12-5.04) |
| Intestinal stenosis | 334 | 24.13 (21.53-27.05) |
| Anal fistula | 327 | 17.86 (15.94-20.00) |
| Anal incontinence | 318 | 8.43 (7.53-9.43) |
| Diarrhoea haemorrhagic | 306 | 9.64 (8.59-10.81) |
| Anal fissure | 269 | 14.55 (12.85-16.47) |
| Small intestinal obstruction | 259 | 6.41 (5.67-7.26) |
| Mouth ulceration | 248 | 3.43 (3.02-3.89) |
| Proctalgia | 248 | 12.07 (10.62-13.73) |
| Large intestine polyp | 242 | 8.61 (7.57-9.79) |
| Faeces discoloured | 224 | 3.04 (2.67-3.47) |
| Pancreatitis | 217 | 1.41 (1.23-1.61) |
| Gastrointestinal pain | 185 | 4.60 (3.98-5.33) |
| Abnormal faeces | 178 | 5.87 (5.06-6.82) |
| Inflammatory bowel disease | 176 | 8.42 (7.24-9.79) |
| Large intestinal stenosis | 160 | 21.87 (18.57-25.76) |
| Dyschezia | 154 | 12.69 (10.78-14.94) |
| Irritable bowel syndrome | 137 | 1.84 (1.56-2.18) |
| Intestinal perforation | 131 | 3.48 (2.92-4.13) |
| Ileal stenosis | 120 | 21.97 (18.18-26.54) |
| Proctitis | 112 | 11.68 (9.65-14.13) |
| Rectal discharge | 105 | 14.09 (11.56-17.19) |
| Abdominal mass | 105 | 12.28 (10.08-14.96) |
| Abdominal hernia | 104 | 6.43 (5.29-7.81) |
| Faeces hard | 103 | 7.25 (5.95-8.82) |
| Anorectal discomfort | 98 | 7.77 (6.35-9.52) |
| Food poisoning | 97 | 5.08 (4.15-6.22) |
| Abdominal adhesions | 92 | 8.36 (6.78-10.3) |
| Large intestinal ulcer | 86 | 10.09 (8.12-12.53) |
| Aphthous ulcer | 85 | 2.44 (1.97-3.03) |
| Colitis microscopic | 83 | 4.43 (3.56-5.51) |
| Rectal tenesmus | 82 | 21.22 (16.89-26.67) |
| Anal haemorrhage | 81 | 8.70 (6.96-10.87) |
| Bowel movement irregularity | 78 | 2.58 (2.06-3.23) |
| Pouchitis | 77 | 31.56 (24.79-40.18) |
| Intestinal fistula | 76 | 16.38 (12.96-20.71) |
| Intestinal haemorrhage | 75 | 3.90 (3.11-4.91) |
| Haemorrhoidal haemorrhage | 73 | 4.03 (3.20-5.08) |
| Inguinal hernia | 69 | 3.04 (2.40-3.86) |
| Umbilical hernia | 68 | 3.95 (3.11-5.02) |
| Gastrointestinal scarring | 65 | 27.86 (21.47-36.15) |
| Enteritis | 63 | 2.63 (2.05-3.37) |
| Ileus | 62 | 1.63 (1.27-2.09) |
| Intestinal ulcer | 61 | 11.66 (9.01-15.11) |
| Gastrointestinal obstruction | 60 | 6.65 (5.14-8.61) |
| Gastrointestinal sounds abnormal | 56 | 3.31 (2.54-4.31) |
| Abdominal tenderness | 55 | 3.50 (2.68-4.57) |
| Large Intestine perforation | 53 | 2.31 (1.76-3.03) |
| Gastrointestinal motility disorder | 53 | 2.65 (2.02-3.48) |
| Small Intestinal stenosis | 50 | 13.61 (10.22-18.14) |
| Intestinal mass | 46 | 9.42 (7.00-12.66) |
| Ileal ulcer | 43 | 12.52 (9.20-17.05) |
| Abdominal rigidity | 40 | 4.33 (3.17-5.93) |
| Intestinal polyp | 40 | 6.93 (5.05-9.50) |
| Proctitis ulcerative | 36 | 24.72 (17.46-34.99) |
| Diverticulum intestinal | 33 | 2.30 (1.63-3.24) |
| Colon dysplasia | 32 | 30.40 (20.93-44.17) |
| Infrequent bowel movements | 32 | 3.57 (2.52-5.07) |
| Enterocutaneous fistula | 31 | 11.21 (7.81-16.11) |
| Faecaloma | 30 | 1.66 (1.16-2.38) |
| Anal stenosis | 30 | 18.38 (12.64-26.75) |
| Subileus | 29 | 4.41 (3.05-6.38) |
| Anorectal disorder | 27 | 4.23 (2.89-6.20) |
| Gastrointestinal fistula | 26 | 7.84 (5.29-11.60) |
| Volvulus | 25 | 2.93 (1.97-4.35) |
| Rectal prolapse | 25 | 5.95 (3.99-8.85) |
| Rectal stenosis | 25 | 22.61 (14.93-34.24) |
| Short-bowel syndrome | 24 | 10.03 (6.65-15.13) |
| Gastrointestinal wall thickening | 24 | 3.79 (2.53-5.68) |
| Gastrointestinal polyp | 24 | 18.06 (11.88-27.45) |
| Rectal polyp | 23 | 7.02 (4.63-10.65) |
| Rectal ulcer | 23 | 5.92 (3.91-8.96) |
| Large intestinal haemorrhage | 23 | 2.82 (1.87-4.25) |
| Enterovesical fistula | 23 | 10.09 (6.63-15.34) |
| Gastrointestinal ulcer | 22 | 3.09 (2.03-4.70) |
| Small intestinal perforation | 21 | 3.40 (2.21-5.24) |
| Gastric polyps | 21 | 2.69 (1.75-4.15) |
| Immune-mediated enterocolitis | 20 | 1.59 (1.02-2.46) |
| Appendix disorder | 19 | 4.12 (2.61-6.49) |
| Large intestinal obstruction | 19 | 2.15 (1.37-3.38) |
| Anal pruritus | 19 | 4.84 (3.07-7.63) |
| Colonic fistula | 18 | 9.48 (5.91-15.23) |
| Rectal fissure | 18 | 8.96 (5.58-14.37) |
| Anal inflammation | 17 | 6.76 (4.17-10.97) |
| Chronic gastritis | 17 | 1.69 (1.05-2.72) |
| Bile acid malabsorption | 17 | 18.81 (11.43-30.97) |
| Megacolon | 16 | 3.24 (1.97-5.30) |
| Intra-abdominal fluid collection | 16 | 1.98 (1.21-3.24) |
| Gastrointestinal mucosa hyperaemia | 16 | 12.59 (7.59-20.89) |
| Terminal ileitis | 15 | 6.88 (4.11-11.52) |
| Gastrointestinal tract irritation | 15 | 3.40(2.04-5.67) |
| Autoimmune pancreatitis | 14 | 7.34 (4.30-12.51) |
| Anal ulcer | 14 | 4.95 (2.91-8.42) |
| Precancerous lesion of digestive tract | 14 | 35.11 (19.84-62.13) |
| Intussusception | 13 | 3.96 (2.28-6.85) |
| Fistula of small intestine | 13 | 7.69 (4.42-13.39) |
| Gastrointestinal stenosis | 12 | 7.43 (4.17-13.22) |
| Anal skin tags | 12 | 22.80 (12.52-41.51) |
| Enterocolonic fistula | 11 | 31.83 (16.8-60.31) |
| Duodenal stenosis | 10 | 6.99 (3.72-13.15) |
| Stomach mass | 10 | 3.14 (1.68-5.86) |
| Small intestine ulcer | 10 | 4.95 (2.64-9.27) |
| Intestinal dilatation | 9 | 2.22 (1.15-4.28) |
| Haemorrhoids thrombosed | 9 | 6.51 (3.35-12.66) |
| Intestinal prolapse | 9 | 6.20 (3.19-12.05) |
| Anorectal swelling | 9 | 9.30 (4.76-18.17) |
| Large intestinal ulcer haemorrhage | 8 | 7.52 (3.71-15.25) |
| Ileal perforation | 8 | 4.26 (2.12-8.59) |
| Abdominal hernia obstructive | 7 | 15.13 (7.01-32.69) |
| Rectal spasm | 7 | 8.33 (3.91-17.77) |
| Anal fissure haemorrhage | 7 | 9.21 (4.31-19.66) |
| Mechanical iIleus | 7 | 3.31 (1.57-6.98) |
| Pseudopolyposis | 7 | 15.49 (7.17-33.48) |
| Anal cyst | 7 | 32.92 (14.75-73.48) |
| Gastroenteritis eosinophilic | 7 | 6.04 (2.85-12.82) |
| Gastrointestinal dysplasia | 6 | 14.85 (6.47-34.1) |
| Gastrointestinal mucosal disorder | 6 | 2.97 (1.33-6.65) |
| Salivary duct obstruction | 5 | 11.76 (4.76-29.02) |
| Rectal dysplasia | 5 | 52.25 (19.40-140.73) |
| Gastrointestinal tract mucosal Pigmentation | 5 | 5.47 (2.25-13.30) |
| Anal erythema | 5 | 10.81 (4.39-26.63) |
| Anal spasm | 4 | 17.10 (6.14-47.59) |
| Jejunal stenosis | 4 | 14.75 (5.33-40.82) |
| Intestinal cyst | 4 | 3.67 (1.36-9.87) |
| Epiploic appendagitis | 4 | 9.52 (3.49-26.01) |
| Internal hernia | 4 | 7.30 (2.69-19.83) |
| Oesophageal food impaction | 4 | 3.15 (1.17-8.46) |
| Anorectal stenosis | 4 | 27.87 (9.75-79.64) |
| Intestinal fibrosis | 4 | 4.05 (1.50-10.89) |
| Small intestine polyp | 4 | 11.76 (4.28-32.28) |
| Cryptitis | 4 | 6.43 (2.37-17.42) |
| Anal blister | 4 | 31.35 (10.88-90.35) |
| Large intestine erosion | 4 | 8.75 (3.21-23.84) |
| Gastric mucosal hypertrophy | 3 | 3.62 (1.15-11.34) |
| Rectal ulcer haemorrhage | 3 | 3.38 (1.08-10.58) |
| Acquired oesophageal web | 3 | 3.38 (1.08-10.58) |
| Intestinal strangulation | 3 | 5.32 (1.69-16.77) |
| Cyclic vomiting syndrome | 3 | 3.26 (1.04-10.21) |
| Incarcerated umbilical hernia | 3 | 7.52 (2.37-23.86) |
| Volvulus of Small bowel | 3 | 4.44 (1.41-13.96) |
| Abdominal incarcerated hernia | 3 | 13.76 (4.26-44.45) |
| Anal polyp | 3 | 6.88 (2.17-21.78) |
| Duodenogastric reflux | 3 | 3.36 (1.07-10.52) |
| Lumbar hernia | 3 | 4.82 (1.53-15.17) |
| Anal rash | 3 | 9.56 (3.00-30.51) |

**Abbreviation:** PTs, preferred terms; ROR, reporting odds ratio; CI, confidence interval.

**Supplementary Table S4**

Comparison of clinical characteristics (Serious vs. Non-serious) for all significant gastrointestinal AEs.

| Characteristics | Non-serious cases (N=1,779) | Serious cases (N=16,164) | Statistic  (*χ*²) | *p* value |
| --- | --- | --- | --- | --- |
| **Gender, n (%)** |  |  |  |  |
| Female | 1,026 (9.96) | 9,280 (90.04) | 9.65 | 0.002^a^ |
| Male | 621 (8.56) | 6,637 (91.44) |  |  |
| **Weight (kg), n (%)** |  |  |  |  |
| <50 | 32 (12.70) | 220 (87.30) | 17.38 | <0.001^a^ |
| 50~100 | 274 (17.06) | 1,332 (82.94) |  |  |
| >100 | 44 (28.57) | 110 (72.43) |  |  |
| **Age (years), n (%)** |  |  |  |  |
| <18 | 27 (8.57) | 288 (91.43) | 78.50 | <0.001^a^ |
| 18~45 | 214 (3.88) | 5,303 (96.12) |  |  |
| 46~65 | 197 (4.53) | 4,151 (95.47) |  |  |
| >65 | 236 (8.00) | 2,714 (92.00) |  |  |
| **Types of AEs, n (%)** |  |  |  |  |
| Diarrhoea | 490 (9.04) | 4,930 (90.96) | 6.62^c^ | <0.05^a^ |
| Abdominal pain | 387 (8.11) | 4,383 (91.89) | 0^c^ | 0.28^a^ |
| Nausea | 345 (14.99) | 1,957 (85.01) | 151.57^c^ | <0.05^a^ |
| Haematochezia* | 227 (5.89) | 3,628 (94.11) | 28.29^c^ | <0.05^a^ |
| Frequent bowel movements* | 213 (6.98) | 2,839 (93.02) | 5.73^c^ | 0.13^a^ |
| Vomiting | 160 (10.26) | 1,399 (89.74) | 9.48^c^ | <0.05^a^ |
| Constipation | 125 (9.38) | 1,207 (90.62) | 2.69^c^ | 0.02^a^ |
| Abdominal distension* | 111 (10.31) | 966 (89.69) | 6.66^c^ | <0.05^a^ |
| Abdominal pain upper | 110 (9.08) | 1,102 (90.92) | 1.34^c^ | 0.08^a^ |
| Flatulence | 86 (12.48) | 603 (87.52) | 17.09^c^ | <0.05^a^ |
| Abdominal discomfort | 75 (9.69) | 699 (90.31) | 2.33^c^ | 0.04^a^ |
| Rectal haemorrhage | 75 (5.57) | 1,271 (94.43) | 11.88^c^ | <0.05^a^ |
| Defaecation urgency* | 70 (7.58) | 853 (92.42) | 0.31^c^ | 0.94^a^ |
| Mucous stools* | 52 (5.71) | 858 (94.29) | 6.97^c^ | 0.03^a^ |
| Colitis* | 42 (8.55) | 449 (91.45) | 0.07^c^ | 0.53^a^ |
| Intestinal Obstruction | 42 (4.08) | 987 (95.92) | 22.64^c^ | <0.05^a^ |
| Mouth ulceration* | 38 (15.32) | 210 (84.68) | 16.27^c^ | <0.05^a^ |
| Abdominal pain lower | 30 (7.67) | 361 (92.33) | 0.06^c^ | 1^a^ |
| Anal incontinence* | 26 (8.18) | 292 (91.82) | 0^c^ | 0.83^a^ |
| Anal fistula | 23 (7.03) | 304 (92.97) | 0.4^c^ | 0.73^a^ |
| Dyspepsia | 23 (7.69) | 276 (92.31) | 0.03^c^ | 1^a^ |
| Faeces soft* | 22 (3.99) | 530 (96.01) | 12.34^c^ | <0.05^a^ |
| Gastrointestinal inflammation* | 22 (5.57) | 373 (94.43) | 3.18^c^ | 0.13^a^ |
| Intestinal stenosis | 22 (6.59) | 312 (93.41) | 0.88^c^ | 0.51^a^ |
| Stomatitis | 22 (19.64) | 90 (80.36) | 18.37^c^ | <0.05^a^ |
| Faeces discoloured* | 21 (9.38) | 203 (90.62) | 0.31^c^ | 0.41^a^ |
| Gastrointestinal pain* | 21 (11.35) | 164 (88.65) | 2.15^c^ | 0.08^a^ |
| Haemorrhoids* | 21 (5.29) | 376 (94.71) | 3.97^c^ | 0.09^a^ |
| Lip swelling | 21 (50.00) | 21 (50.00) | - | <0.001^a^ |
| Gastrointestinal disorder | 19 (5.85) | 306 (94.15) | 2.00^c^ | 0.25^a^ |
| Dysphagia | 17 (19.54) | 70 (80.46) | 13.67^c^ | <0.05^a^ |
| Gastrooesophageal reflux disease | 17 (7.36) | 214 (92.64) | 0.1^c^ | 0.94^a^ |
| Proctalgia | 16 (6.45) | 232 (93.55) | 0.74^c^ | 0.54^a^ |
| Diarrhoea haemorrhagic* | 15 (4.90) | 291 (95.10) | 3.89^c^ | 0.08^a^ |
| Inflammatory bowel disease* | 14 (7.95) | 162 (92.05) | 0^c^ | 1^a^ |
| Oral pain | 14 (41.18) | 20 (58.82) | - | <0.05^a^ |
| Proctitis | 13 (11.61) | 99 (88.39) | 1.37^c^ | 0.17^a^ |
| Abnormal faeces* | 12 (6.74) | 166 (93.26) | 0.3^c^ | 0.73^a^ |
| Large intestine polyp* | 12 (4.96) | 230 (95.04) | 2.88^c^ | 0.14^a^ |
| Dry mouth | 11 (15.49) | 60 (84.51) | 4.21^c^ | 0.02^a^ |
| Small intestinal obstruction | 11 (4.25) | 248 (95.75) | 4.76^c^ | 0.05^a^ |
| Anal fissure | 10 (3.72) | 259 (96.28) | 6.49^c^ | 0.02^a^ |
| Irritable bowel syndrome* | 10 (7.30) | 127 (92.70) | 0.04^c^ | 0.99^a^ |
| Anorectal discomfort* | 9 (9.18) | 89 (90.82) | 0.04^c^ | 0.72^a^ |
| Bowel movement irregularity* | 9 (11.54) | 69 (88.46) | 0.8^c^ | 0.29^a^ |
| Faeces hard* | 9 (8.74) | 94 (91.26) | 0^c^ | 0.83^a^ |
| Hypoaesthesia oral | 9 (27.27) | 24 (72.73) | 0^c^ | <0.05^a^ |
| Dyschezia* | 8 (5.19) | 146 (94.81) | 1.42^c^ | 0.31^a^ |
| Food poisoning* | 8 (8.25) | 89 (91.75) | 0^c^ | 0.99^a^ |
| Inguinal hernia* | 8 (11.59) | 61 (88.41) | 0.69^c^ | 0.32^a^ |
| Paraesthesia oral | 8 (28.57) | 20 (71.43) | - | <0.05^a^ |
| Toothache | 8 (14.29) | 48 (85.71) | - | 0.08^a^ |
| Umbilical hernia | 8 (11.76) | 60 (88.24) | 0.76^c^ | 0.3^a^ |
| Aphthous ulcer* | 7 (8.24) | 78 (91.76) | 0^c^ | 1^a^ |
| Gastritis | 7 (7.45) | 87 (92.55) | 0^c^ | 1^a^ |
| Haematemesis | 7 (13.73) | 44 (86.27) | - | 0.11^b^ |
| Haemorrhoidal haemorrhage* | 7 (9.59) | 66 (90.41) | 0.06^c^ | 0.7^a^ |
| Pouchitis* | 7 (9.09) | 70 (90.91) | 0.01^c^ | 0.81^a^ |
| Abdominal hernia* | 6 (5.77) | 98 (94.23) | 0.5^c^ | 0.58^a^ |
| Large intestinal stenosis* | 6 (3.75) | 154 (96.25) | 3.57^c^ | 0.08^a^ |
| Rectal discharge* | 6 (5.71) | 99 (94.29) | 0.53^c^ | 0.56^a^ |
| Retching | 6 (16.22) | 31 (83.78) | - | 0.06^b^ |
| Swollen tongue | 6 (11.76) | 45 (88.24) | - | 0.28^b^ |
| Abdominal adhesions* | 5 (5.43) | 87 (94.57) | 0.57^c^ | 0.54^a^ |
| Abdominal tenderness* | 5 (9.09) | 50 (90.91) | - | 0.61^b^ |
| Coeliac disease | 5 (18.52) | 22 (81.48) | - | 0.05^b^ |
| Eructation | 5 (15.62) | 27 (84.38) | - | 0.1^b^ |
| Gastrointestinal motility disorder* | 5 (9.43) | 48 (90.57) | - | 0.6^b^ |
| Gastrointestinal obstruction* | 5 (8.33) | 55 (91.67) | - | 0.81^b^ |
| Gastrointestinal sounds abnormal* | 5 (8.93) | 51 (91.07) | - | 0.62^b^ |
| Pancreatitis | 5 (2.30) | 212 (97.70) | 9.16^c^ | <0.05^a^ |
| rectal tenesmus* | 5 (6.10) | 77 (93.90) | 0.22^c^ | 0.74^a^ |
| Abdominal rigidity* | 4 (10.00) | 36 (90.00) | - | 0.55^b^ |
| Cheilitis | 4 (36.36) | 7 (63.64) | - | 0.01^b^ |
| Enteritis | 4 (6.35) | 59 (93.65) | 0.08 | 1^b^ |
| Gastrointestinal haemorrhage | 4 (3.36) | 115 (96.64) | 3.03^c^ | 0.11^a^ |
| Gastrointestinal scarring* | 4 (6.15) | 61 (93.85) | 0.13^c^ | 0.81^a^ |
| Gastrointestinal ulcer* | 4 (18.18) | 18 (81.82) | - | 0.08^b^ |
| Gingival bleeding | 4 (36.36) | 7 (63.64) | - | 0.01^b^ |
| Gingival swelling | 4 (44.44) | 5 (55.56) | - | <0.05^b^ |
| Impaired gastric emptying | 4 (17.39) | 19 (82.61) | - | 0.1^b^ |
| Infrequent bowel movements* | 4 (12.5) | 28 (87.5) | - | 0.31^b^ |
| Intestinal haemorrhage* | 4 (5.33) | 71 (94.67) | 0.46^c^ | 0.58^a^ |
| Intestinal perforation* | 4 (3.05) | 127 (96.95) | 3.89^c^ | 0.07^a^ |
| Large intestine perforation | 4 (7.55) | 49 (92.45) | - | 1^b^ |
| Lower gastrointestinal haemorrhage | 4 (18.18) | 18 (81.82) | - | 0.08^b^ |
| Mouth swelling | 4 (36.36) | 7 (63.64) | - | 0.01^b^ |
| Anal haemorrhage* | 3 (3.70) | 78 (96.30) | 1.58^c^ | 0.25^a^ |
| dental caries | 3 (20.00) | 12 (80.00) | - | 0.1^b^ |
| Diverticulum | 3 (17.65) | 14 (82.35) | - | 0.14^b^ |
| gastric ulcer | 3 (4.84) | 59 (95.16) | 0.52^d^ | 0.63^b^ |
| Gastrointestinal tract irritation | 3 (20.00) | 12 (80.00) | - | 0.1^b^ |
| Gingival pain | 3 (8.82) | 31 (91.18) | - | 0.74^b^ |
| Ileal stenosis | 3 (2.50) | 117 (97.50) | 4.39^c^ | 0.05^a^ |
| Ileus | 3 (4.84) | 59 (95.16) | 0.52^d^ | 0.63^b^ |
| Intestinal fistula* | 3 (3.95) | 73 (96.05) | 1.27^c^ | 0.31^a^ |
| Intestinal ulcer* | 3 (4.92) | 58 (95.08) | - | 0.63^b^ |
| Lip dry | 3 (30.00) | 7 (70.00) | - | 0.04^b^ |
| Malabsorption | 3 (15.00) | 17 (85.00) | - | 0.2^b^ |
| oral discomfort | 3 (17.65) | 14 (82.35) | - | 0.14^b^ |
| Rectal prolapse* | 3 (12.00) | 22 (88.00) | - | 0.44^b^ |
| Rectal ulcer* | 3 (13.04) | 20 (86.96) | - | 0.42^b^ |
| Tongue discomfort | 3 (30.00) | 7 (70.00) | - | 0.04^b^ |
| Tongue ulceration | 3 (17.65) | 14 (82.35) | - | 0.14^b^ |
| Anal inflammation | 2 (11.76) | 15 (88.24) | - | 0.38^b^ |
| Ascites | 2 (3.45) | 56 (96.55) | - | 0.32^b^ |
| Chapped lips | 2 (28.57) | 5 (71.43) | - | 0.1^b^ |
| Colitis microscopic* | 2 (2.41) | 81 (97.59) | 2.92^c^ | 0.11^a^ |
| Faecaloma* | 2 (6.67) | 28 (93.33) | - | 1^b^ |
| Gastric dilatation | 2 (40.00) | 3 (60.00) | - | 0.05^b^ |
| Gastric haemorrhage | 2 (12.50) | 14 (87.50) | - | 0.35^b^ |
| Gastrointestinal wall thickening* | 2 (8.33) | 22 (91.67) | - | 0.71^b^ |
| Gingival erythema | 2 (40.00) | 3 (60.00) | - | 0.05^b^ |
| Glossodynia | 2 (14.29) | 12 (85.71) | - | 0.29^b^ |
| Large intestinal Haemorrhage* | 2 (8.70) | 21 (91.30) | - | 0.7^b^ |
| Large intestinal ulcer* | 2 (2.33) | 84 (97.67) | 3.15^c^ | 0.1^a^ |
| Lip oedema | 2 (40.00) | 3 (60.00) | - | 0.05^b^ |
| Melaena | 2 (3.33) | 58 (96.67) | - | 0.33^b^ |
| Oesophageal pain | 2 (14.29) | 12 (85.71) | - | 0.29^b^ |
| Oesophageal stenosis | 2 (18.18) | 9 (81.82) | - | 0.21^b^ |
| Oesophageal ulcer | 2 (13.33) | 13 (86.67) | - | 0.32^b^ |
| Oral disorder | 2 (15.38) | 11 (84.62) | - | 0.26^b^ |
| Oral lichen planus | 2 (50.00) | 2 (50.00) | - | 0.03^b^ |
| Oral mucosal blistering | 2 (25.00) | 6 (75.00) | - | 0.12^b^ |
| Oral mucosal eruption | 2 (40.00) | 3 (60.00) | - | 0.05^b^ |
| Oral pruritus | 2 (50.00) | 2 (50.00) | - | 0.03^b^ |
| Precancerous lesion of digestive tract | 2 (14.29) | 12 (85.71) | - | 0.29^b^ |
| Proctitis ulcerative* | 2 (5.56) | 34 (94.44) | - | 1^b^ |
| Rectal polyp* | 2 (8.70) | 21 (91.30) | - | 0.7^b^ |
| Small intestinal perforation* | 2 (9.52) | 19 (90.48) | - | 0.67^b^ |
| Tongue coated | 2 (66.67) | 1 (33.33) | - | 0.02^b^ |
| Tongue discolouration | 2 (40.00) | 3 (60.00) | - | 0.05^b^ |
| Tongue disorder | 2 (18.18) | 9 (81.82) | - | 0.21^b^ |
| Tooth discolouration | 2 (50.00) | 2 (50.00) | - | 0.03^b^ |
| Tooth disorder | 2 (5.88) | 32 (94.12) | - | 1^b^ |
| Abdominal mass* | 1 (0.95) | 104 (99.05) | 6.34^c^ | 0.02^a^ |
| Acute oesophageal mucosal lesion | 1 (50.00) | 1 (50.00) | - | 0.15^b^ |
| Anal cyst | 1 (14.29) | 6 (85.71) | - | 0.43^b^ |
| Anal pruritus | 1 (5.26) | 18 (94.74) | - | 1^b^ |
| Anal rash | 1 (33.33) | 2 (66.67) | - | 0.21^b^ |
| Anal spasm | 1 (25.00) | 3 (75.00) | - | 0.27^b^ |
| Anal stenosis* | 1 (3.33) | 29 (96.67) | - | 0.73^b^ |
| Angular cheilitis | 1 (20.00) | 4 (80.00) | - | 0.33^b^ |
| Anorectal swelling | 1 (11.11) | 8 (88.89) | - | 0.51^b^ |
| Autoimmune pancreatitis | 1 (7.14) | 13 (92.86) | - | 1^b^ |
| Bile acid malabsorption | 1 (5.88) | 16 (94.12) | - | 1^b^ |
| Colon dysplasia | 1 (3.12) | 31 (96.88) | - | 0.51^b^ |
| Defaecation disorder | 1 (25.00) | 3 (75.00) | - | 0.27^b^ |
| Dental discomfort | 1 (50.00) | 1 (50.00) | - | 0.15^b^ |
| Diverticulum intestinal* | 1 (3.03) | 32 (96.97) | - | 0.51^b^ |
| Dysbiosis | 1 (12.50) | 7 (87.50) | - | 0.47^b^ |
| Enlarged uvula | 1 (50.00) | 1 (50.00) | - | 0.15^b^ |
| Enterocolonic fistula | 1 (9.09) | 10 (90.91) | - | 0.59^b^ |
| Enterocutaneous fistula* | 1 (3.23) | 30 (96.77) | - | 0.51^b^ |
| Enterovesical fistula | 1 (4.35) | 22 (95.65) | - | 1^b^ |
| Epigastric discomfort | 1 (8.33) | 11 (91.67) | - | 1^b^ |
| Functional gastrointestinal disorder | 1 (8.33) | 11 (91.67) | - | 1^b^ |
| Gastric mucosal hypertrophy | 1 (33.33) | 2 (66.67) | - | 0.21^b^ |
| Gastric mucosal lesion | 1 (33.33) | 2 (66.67) | - | 0.21^b^ |
| Gastric stenosis | 1 (50.00) | 1 (50.00) | - | 0.15^b^ |
| Gastrointestinal erosion | 1 (25.00) | 3 (75.00) | - | 0.27^b^ |
| Gastrointestinal fistula* | 1 (3.85) | 25 (96.15) | - | 0.72^b^ |
| Gingival recession | 1 (33.33) | 2 (66.67) | - | 0.21^b^ |
| Hiatus hernia | 1 (2.44) | 40 (97.56) | - | 0.37^b^ |
| Ileus paralytic | 1 (9.09) | 10 (90.91) | - | 0.59^b^ |
| Immune-mediated Enterocolitis | 1 (5.00) | 19 (95.00) | - | 1^b^ |
| Incarcerated umbilical hernia | 1 (33.33) | 2 (66.67) | - | 0.21^b^ |
| Intestinal dilatation | 1 (11.11) | 8 (88.89) | - | 0.51^b^ |
| Intestinal polyp* | 1 (2.50) | 39 (97.50) | - | 0.37^b^ |
| Large intestine erosion | 1 (25.00) | 3 (75.00) | - | 0.27^b^ |
| Leukoplakia oral | 1 (50.00) | 1 (50.00) | - | 0.15^b^ |
| Lip blister | 1 (25.00) | 3 (75.00) | - | 0.27^b^ |
| Lip erythema | 1 (33.33) | 2 (66.67) | - | 0.21^b^ |
| Lip pain | 1 (25.00) | 3 (75.00) | - | 0.27^b^ |
| Lumbar hernia | 1 (33.33) | 2 (66.67) | - | 0.21^b^ |
| Mouth cyst | 1 (50.00) | 1 (50.00) | - | 0.15^b^ |
| Odynophagia | 1 (10.00) | 9 (90.00) | - | 0.55^b^ |
| Oral mucosal exfoliation | 1 (33.33) | 2 (66.67) | - | 0.21^b^ |
| Palatal swelling | 1 (50.00) | 1 (50.00) | - | 0.15^b^ |
| Pancreatic disorder | 1 (6.67) | 14 (93.33) | - | 1^b^ |
| Pancreatitis acute | 1 (1.52) | 65 (98.48) | 3.04^c^ | 0.1^a^ |
| Pancreatitis necrotising | 1 (50.00) | 1 (50.00) | - | 0.15^b^ |
| Rectal fissure | 1 (5.56) | 17 (94.44) | - | 1^b^ |
| Rectal perforation | 1 (25.00) | 3 (75.00) | - | 0.27^b^ |
| Salivary duct obstruction | 1 (20.00) | 4 (80.00) | - | 0.33^b^ |
| Small intestinal stenosis | 1 (2.00) | 49 (98.00) | - | 0.18^b^ |
| Steatorrhoea | 1 (10.00) | 9 (90.00) | - | 0.55^b^ |
| Stomach mass | 1 (10.00) | 9 (90.00) | - | 0.55^b^ |
| Terminal ileitis | 1 (6.67) | 14 (93.33) | - | 1^b^ |
| Tooth loss | 1 (14.29) | 6 (85.71) | - | 0.43^b^ |
| Vomiting projectile | 1 (25.00) | 3 (75.00) | - | 0.27^b^ |

**Note:** ^a^ Proportions were compared using Pearson’s chi-squared (***χ***^2^) test; ^b^ Proportions were compared using Fisher’s exact test; ^c^ The ***χ***^2^ statistic of the Pearson’s chi-squared (***χ***^2^) test; ^d^ The Fisher’s exact statistic of the Fisher’s exact test. * Emerging findings of vedolizumab-associated gastrointestinal AEs from FAERS database.

**Abbreviation:** AEs, adverse events; n, number of cases.

**Supplementary Table S5**

Clinical relevance assessment for Low Relevance Signals (Score 0~2).

| PTs | N | ROR_025_ | Death (n) | IMEs/DMEs | Relevance level (score) |
| --- | --- | --- | --- | --- | --- |
| Constipation | 1,332 | 1.60 | 13 | NA | Low (1) |
| Abdominal pain upper | 1,212 | 1.56 | 15 | NA | Low (1) |
| Abdominal distension | 1,077 | 2.78 | 7 | NA | Low (2) |
| Defaecation urgency * | 923 | 39.81 | 4 | NA | Low (2) |
| Mucous stools * | 910 | 57.70 | 0 | NA | Low (2) |
| Abdominal discomfort | 774 | 1.08 | 4 | NA | Low (1) |
| Flatulence | 689 | 3.34 | 6 | NA | Low (2) |
| Faeces soft * | 552 | 17.41 | 3 | NA | Low (2) |
| Haemorrhoids * | 397 | 5.69 | 2 | NA | Low (2) |
| Gastrointestinal Inflammation * | 395 | 12.29 | 3 | NA | Low (2) |
| Abdominal pain lower | 391 | 4.12 | 1 | NA | Low (2) |
| Anal fistula | 327 | 15.94 | 1 | NA | Low (2) |
| Anal incontinence * | 318 | 7.53 | 6 | NA | Low (2) |
| Anal fissure | 269 | 12.85 | 1 | NA | Low (2) |
| Mouth ulceration * | 248 | 3.02 | 0 | NA | Low (2) |
| Proctalgia | 248 | 10.62 | 0 | NA | Low (2) |
| Large intestine polyp * | 242 | 7.57 | 1 | NA | Low (2) |
| Faeces discoloured * | 224 | 2.67 | 4 | NA | Low (2) |
| Pancreatitis* | 217 | 1.23 | 3 | NA | Low (2) |
| Gastrointestinal pain * | 185 | 3.98 | 3 | NA | Low (2) |
| Abnormal faeces * | 178 | 5.06 | 0 | NA | Low (2) |
| Dyschezia * | 154 | 10.78 | 0 | NA | Low (2) |
| Irritable bowel syndrome * | 137 | 1.56 | 1 | NA | Low (1) |
| Proctitis | 112 | 9.65 | 0 | NA | Low (2) |
| Rectal discharge * | 105 | 10.08 | 0 | NA | Low (2) |
| Abdominal mass * | 105 | 11.56 | 1 | NA | Low (2) |
| Abdominal hernia * | 104 | 5.29 | 0 | NA | Low (2) |
| Faeces hard * | 103 | 5.95 | 0 | NA | Low (2) |
| Anorectal discomfort * | 98 | 6.35 | 0 | NA | Low (2) |
| Food poisoning * | 97 | 4.15 | 1 | NA | Low (2) |
| Abdominal adhesions * | 92 | 6.78 | 4 | NA | Low (2) |
| Aphthous ulcer * | 85 | 1.97 | 1 | NA | Low (2) |
| Rectal tenesmus * | 82 | 16.89 | 0 | NA | Low (2) |
| Anal haemorrhage * | 81 | 6.96 | 1 | NA | Low (2) |
| Bowel movement Irregularity * | 78 | 2.06 | 0 | NA | Low (2) |
| Pouchitis * | 77 | 24.79 | 0 | NA | Low (2) |
| Haemorrhoidal Haemorrhage * | 73 | 3.20 | 0 | NA | Low (2) |
| Inguinal hernia * | 69 | 2.40 | 0 | NA | Low (2) |
| Umbilical hernia | 68 | 3.11 | 0 | NA | Low (2) |
| Gastrointestinal scarring * | 65 | 21.47 | 1 | NA | Low (2) |
| Enteritis | 63 | 2.05 | 2 | NA | Low (2) |
| Ileus | 62 | 1.27 | 0 | IME | Low (2) |
| Gastrointestinal sounds abnormal * | 56 | 2.54 | 0 | NA | Low (2) |
| Abdominal tenderness * | 55 | 2.68 | 0 | NA | Low (2) |
| Gastrointestinal motility disorder * | 53 | 1.76 | 0 | NA | Low (2) |
| Intestinal mass * | 46 | 7.00 | 3 | NA | Low (2) |
| Abdominal rigidity * | 40 | 3.17 | 0 | NA | Low (2) |
| Intestinal polyp * | 40 | 5.05 | 0 | NA | Low (2) |
| Diverticulum intestinal * | 33 | 1.63 | 0 | NA | Low (2) |
| Infrequent bowel movements * | 32 | 2.52 | 0 | NA | Low (2) |
| Faecaloma * | 30 | 1.16 | 0 | IME | Low (2) |
| subileus | 29 | 3.05 | 0 | NA | Low (2) |
| Anorectal disorder * | 27 | 2.89 | 0 | NA | Low (2) |
| Rectal prolapse * | 25 | 14.93 | 0 | NA | Low (2) |
| Short-bowel syndrome * | 24 | 11.88 | 1 | NA | Low (2) |
| Gastrointestinal wall thickening * | 24 | 2.53 | 0 | NA | Low (2) |
| Gastrointestinal polyp * | 24 | 6.65 | 0 | NA | Low (2) |
| Rectal polyp * | 23 | 4.63 | 0 | NA | Low (2) |
| Gastrointestinal ulcer * | 22 | 2.03 | 0 | NA | Low (2) |
| Gastric polyps * | 21 | 1.75 | 0 | NA | Low (2) |

**Note:** * New and unexpected signals, not previously reported in the drug label, emerging findings from FAERS database.

**Abbreviations:** PTs, preferred terms; N, the total number of reported cases for a given PT; ROR_025_, the lower limit of 95% confidence interval of ROR; IMEs, important medical events; DMEs, designated medical events. NA, not applicable (for relevant criteria).
